# Multiplex RNA‐based detection of clinically relevant *MET* alterations in advanced non‐small cell lung cancer

**DOI:** 10.1002/1878-0261.12861

**Published:** 2020-12-07

**Authors:** Cristina Aguado, Cristina Teixido, Ruth Román, Roxana Reyes, Ana Giménez‐Capitán, Elba Marin, Carlos Cabrera, Nuria Viñolas, Sergi Castillo, Silvia Muñoz, Ainara Arcocha, Laura López‐Vilaró, Ivana Sullivan, Erika Aldeguer, Sonia Rodríguez, Irene Moya, Santiago Viteri, Andrés Felipe Cardona, Ramon Palmero, Cristina Sainz, Miguel Mesa‐Guzmán, Maria D. Lozano, Andrés Aguilar‐Hernández, Alejandro Martínez‐Bueno, María González‐Cao, Elena Gonzalvo, William P. J. Leenders, Rafael Rosell, Luis M. Montuenga, Aleix Prat, Miguel A. Molina‐Vila, Noemi Reguart

**Affiliations:** ^1^ Laboratory of Oncology, Pangaea Oncology Quirón Dexeus University Hospital Barcelona Spain; ^2^ Thoracic Oncology Unit Department of Pathology Hospital Clínic Barcelona Spain; ^3^ Translational Genomics and Targeted Therapeutics in Solid Tumors Institut d’Investigacions Biomèdiques August Pi i Sunyer (IDIBAPS) Barcelona Spain; ^4^ Thoracic Oncology Unit Department of Medical Oncology Hospital Clínic Barcelona Spain; ^5^ Dr Rosell Oncology Institute Dexeus University Hospital Quiron Salud Group Barcelona Spain; ^6^ Division of Medical Oncology Hospital General de Granollers Barcelona Spain; ^7^ Department of Pathology Hospital de la Santa Creu i Sant Pau Barcelona Spain; ^8^ Division of Medical Oncology Hospital de la Santa Creu i Sant Pau Barcelona Spain; ^9^ Dr Rosell Oncology Institute Teknon Medical Center Quiron Salud Group Barcelona Spain; ^10^ Foundation for Clinical and Applied Cancer Research‐FICMAC Bogotá Colombia; ^11^ Clinical and Translational Oncology Group Institute of Oncology Clínica del Country Bogotá Colombia; ^12^ Division of Medical Oncology Catalan Institute of Oncology L'Hospitalet Barcelona Spain; ^13^ Center for Applied Medical Research (CIMA) University of Navarra Spain; ^14^ CIBERONC Madrid Spain; ^15^ Department of Thoracic Surgery University of Navarra Pamplona Spain; ^16^ IDISNA Pamplona Spain; ^17^ Department of Pathology, Anatomy and Physiology School of Medicine University of Navarra Pamplona Spain; ^18^ Department of Biochemistry Radboud Institute for Molecular Life Sciences Nijmegen The Netherlands; ^19^ Institut d'Investigació en Ciències de la Salut Germans Trias i Pujol Badalona Spain; ^20^ Universitat Autònoma de Barcelona Bellaterra Spain

**Keywords:** amplification, expression, lung cancer, MET, RNA, skipping

## Abstract

MET inhibitors have shown activity in non‐small‐cell lung cancer patients (NSCLC) with *MET* amplification and exon 14 skipping (*METΔex14)*. However, patient stratification is imperfect, and thus, response rates have varied widely. Here, we studied *MET* alterations in 474 advanced NSCLC patients by nCounter, an RNA‐based technique, together with next‐generation sequencing (NGS), fluorescence in situ hybridization (FISH), immunohistochemistry (IHC), and reverse transcriptase polymerase chain reaction (RT–PCR), exploring correlation with clinical benefit. Of the 474 samples analyzed, 422 (89%) yielded valid results by nCounter, which identified 13 patients (3%) with *MET*Δex14 and 15 patients (3.5%) with very‐high *MET* mRNA expression. These two subgroups were mutually exclusive, displayed distinct phenotypes and did not generally coexist with other drivers. For *MET*Δex14, 3/8 (37.5%) samples positive by nCounter tested negative by NGS. Regarding patients with very‐high *MET* mRNA, 92% had *MET* amplification by FISH and/or NGS. However, FISH failed to identify three patients (30%) with very‐high *MET* RNA expression, among which one received MET tyrosine kinase inhibitor treatment deriving clinical benefit. Our results indicate that quantitative mRNA‐based techniques can improve the selection of patients for MET‐targeted therapies.

## Graphical abstract

We studied *MET* alterations in 474 advanced non‐small‐cell lung cancer (NSCLC) patients by nCounter, an RNA‐based technique. We identified 3% with *MET*Δex14 mRNA and 3.5% with very‐high *MET* mRNA expression, a surrogate of MET amplification. *MET* alterations identified by nCounter correlated with clinical benefit from MET inhibitors. Quantitative mRNA‐based techniques can improve the selection of patients for MET‐targeted therapies.

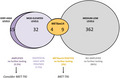

**Abbreviations**
FISH: fluorescence in situ hybridization
HS: histoscore
IHC: immunohistochemistry
nC: nCounter
NGS: next‐generation sequencing
NSCLC: non‐small‐cell lung cancer
RT–PCR: reverse transcription polymerase chain reaction

## 1 Introduction

Aberrant activation of the mesenchymal–epithelial transition (*MET*) gene has recently emerged as an actionable target, particularly in non‐small‐cell lung cancer (NSCLC) [1, 2]. Multiple molecular mechanisms including amplification, point mutations, alternative splicing, and protein overexpression [3, 4] can lead to abnormal *MET* activation, which increases cell proliferation, survival, invasion, and metastasis. *MET* amplification has been described in 1–6% of newly diagnosed NSCLC tumors and constitutes a frequent mechanism of acquired resistance in *EGFR*‐mutant (*EGFR*‐mut) NSCLC patients treated with tyrosine kinase inhibitors (TKI) [5]. *MET* exon 14 alterations in donor and acceptor splicing sites—including point mutations, indels, and whole‐exon deletions—lead to the exclusion (skipping) of *MET* exon 14 at the RNA level (*MET*∆ex14), which has been described in 3–4% of patients with advanced NSCLC [6, 7, 8].

Amplification of the *MET* gene in NSCLC and mutations leading to *MET*∆ex14 were first reported in 2005 and 2006, respectively [9, 10], while two seminal works published in 2015 identified *MET*∆ex14 as a potential therapeutic target in advanced NSCLC [11, 12]. Since then, several trials have evaluated the efficacy of *MET* inhibitors in patients with *MET*∆ex14 and *MET* amplification [13, 14, 15, 16, 17], with response rates varying widely across the different studies. Capmatinib is the first MET inhibitor that has gained recent Food and Drug Administration approval for the treatment of advanced NSCLC with *MET*∆ex14 [18], and novel mechanisms of resistance have meanwhile started to emerge [19].

The most frequent technologies used to assess *MET* gene copy number variations in the clinical setting are fluorescence in situ hybridization (FISH) and next‐generation sequencing (NGS), whereas for *MET*∆ex14 detection, both NGS and reverse transcription polymerase chain reaction (RT–PCR) are commonly used [6, 7, 13, 14, 20, 21, 22, 23, 24]. However, the optimal method(s) and the more adequate thresholds for stratification are not yet defined. The controversies around *MET* testing [25] have been complicated by the small number of comprehensive studies on *MET* status in advanced NSCLC and the fact that most reports evaluating the performance of different techniques have focused on a single *MET* alteration.

The NanoString nCounter™ Analysis System is a high‐throughput, quantitative transcript‐based hybridization technology that allows for the simultaneous analysis of the expression of hundreds of target genes [26] and can be easily incorporated in the routine molecular testing workflow of tumor samples [27]. Although nCounter has been used to determine some *MET* alterations in particular types of tumors [28, 29, 30], it has never been employed for MET testing in NSCLC. In the previous studies, we demonstrated that this methodology can identify relevant gene rearrangements in advanced NSCLC [27, 31]. Here, we aimed to determine whether nCounter could improve the characterization of clinically relevant *MET* alterations. To this end, we screened a large cohort of NSCLC patients and compared the nCounter results with those obtained by standard techniques. Our results indicate that multiplex, RNA‐based techniques such as nCounter have the potential to become the technology of choice to select patients for MET‐targeted therapies.

## 2 Materials and methods

### 2.1 Patients, samples, and cell lines

A total of 474 formalin‐fixed, paraffin‐embedded (FFPE) tumor samples from patients with NSCLC were tested to identify *MET* alterations. Samples were collected from 10 participating hospitals (Supporting Information) with prior full informed patient consent and approval from the corresponding ethical committees. All advanced NSCLC patients arriving to our institutions and having biopsies available with sufficient tumor tissue were offered to participate in the study, which was conducted in accordance with the principles of the Declaration of Helsinki. FFPE slides (4 µm) were obtained by standard procedures and stained with hematoxylin and eosin. A pathologist determined the tumor area and evaluated the percentage of tumor infiltration. RNA was extracted with a high purity FFPE RNA isolation kit (Roche, Mannheim, Germany), while the GeneRead DNA FFPE Kit or the QIAamp DNA FFPE Tissue Kit (Qiagen, Hilden, Germany) was used for DNA extraction from FFPE samples, according to the manufacturer’s instructions. DNA and RNA concentrations were measured by Qubit (Thermo Fisher Scientific, Waltham, MA, USA). Three cell lines (Hs746T, PC9, and E98) were used for validation purposes. The Hs746T cell line, harboring *METΔex14*, was purchased from the American Type Culture Collection. E98 is a patient‐derived astrocytoma cell line with amplification of *MET* gene. *EGFR*‐mut PC9 cells were obtained from F. Hoffman‐La Roche Ltd (Basel, Switzerland) with the authorization of Dr. Mayumi Ono (Kyushu University, Fukuoka, Japan). All cell lines were cultured in RPMI medium with 10% fetal bovine serum under standard conditions and counted after trypsinization. Pellets from a minimum of five T‐75 flasks were used to generate FFPE blocks.

### 2.2 FISH and immunohistochemistry (IHC)

FISH for *MET* was performed with the ZytoLight^®^ SPEC *MET/*centromere 7 (*MET*/CEP7) Dual Color Probe (ZytoVision, Bremerhaven, Germany) according to manufacturer’s instructions. Three positivity criteria for *MET* amplification were used as follows: (a) a ratio (*r*) *MET*/CEP7 ≥ 2; (b) gene copy number (GCN) per cell ≥ 6; (c) ≥ 5 copies in ≥ 50% of cells; (d) or ≥ 15 copies in > 10% tumor cells. These three criteria have been employed for patient stratification in clinical trials of anti‐MET therapies (Table S1). Immunostaining was performed with MET SP44 clone (Roche) on a BenchMark ULTRA automated tissue staining system (Ventana Medical Systems, Tucson, AZ, USA). Two different cutoff points for IHC positivity were considered: (i) membrane intense staining (3+) in ≥ 50% of the tumor cells and (ii) histoscore (HS) ≥ 220.

### 2.3 NGS sample preparation, sequencing run, and data processing

DNA NGS was performed with the GeneRead^®^ QIAact Lung DNA UMI Panel (Qiagen) or OncomineTM Solid Tumour (OST) DNA Panel (Thermo Fisher Scientific), according to the manufacturer’s instructions (Table S2). Both panels target genes frequently altered in lung cancer, including DNA alterations at exons 13–15 of MET and the surrounding intronic regions. The GeneRead panel can also detect amplifications in five genes. For the GeneRead panel, up to 40 ng of purified DNA was used as a template. Clonal amplification was performed on 625 pg of pooled libraries, and, following bead enrichment, the GeneReader instrument was used for sequencing.

RNA‐NGS was performed with the GeneRead^®^ QIAact Lung RNA Fusion UMI Panel (Qiagen) according to the manufacturer’s instructions. This panel targets a fusion‐specific and splicing variants (Table S2), including *MET*Δex14. The GeneRead^®^ QIAact Lung RNA Fusion UMI Panel is designed to enrich selected fusion targets starting with 100 ng of total RNA. After target enrichment and library preparation, clonal amplification was performed using 625 pg of pooled libraries, and, following bead enrichment, the GeneReader instrument was used for sequencing.

Qiagen Clinical Insight Analyze (qci‐a) software was employed to align the read data and call sequence variants, which were imported into the Qiagen Clinical Insight Interpret (QCI‐I) web interface for data interpretation and generation of final custom report. In the case of the OST panel, 10 ng of purified DNA was used as a template. Libraries were pooled at 20 pM and, following ion spheres, sequenced using the Ion Personal Genome Machine (Thermo Fisher Scientific). The Ion Reporter Server (Thermo Fisher Scientific) was used to align the read data to the human reference genome and call sequence variants. The *MET* gene copy numbers by NGS were assessed using the QCI‐A and QCI‐I software. Copy numbers provided by QCI‐I were selected according to the tumor infiltration of the sample, as assessed by an expert pathologist. The GCN ≥ 6 was chosen as a threshold based on the previous experience of our laboratory. During the validation of the NGS panel prior to its implementation in the clinical setting, this threshold had been found to show the highest correlation with FISH *MET*/CEP7 > 2.

### 2.4 **RT**–**PCR analysis for *MET*Δex14 transcripts**

RNA was converted to cDNA using M‐MLV retrotranscriptase (Thermo Fisher Scientific) and oligo‐dT primers, and *MET*Δex14 was amplified using HotStart Taq polymerase (Qiagen) in a 20 µL reaction and visualized in agarose gels. Primers used were located in exons 13 and 15, sequences were as follows: forward (exon 13) 5′‐TTTTCCTGTGGCTGAAAAAGA‐3′ and reverse (exon 15) 5′‐GGGGACATGTCTGTCAGAGG‐3′. Amplification generated a 246‐bp band for wild‐type (wt) MET RNA and a 106‐bp band for METΔex14. Positive samples were confirmed by bidirectional Sanger sequencing of RT–PCR products, using the big‐dye 3.1 sequencing kit (Applied Biosystems, Waltham, MA, USA).

### 2.5 nCounter analysis

Total RNA was hybridized with a custom‐designed mixture of biotinylated capture tags and fluorescently labeled reporter probes (Elements Chemistry) that included, among others, probes for *MET*‐wt and *MET*Δex14 target sequences. Detailed sequence information for the *MET* gene target regions is provided in Table S3. The codeset also included probes for housekeeping genes (actin beta, *ACTB*; proteasome 26S subunit ATPase 4, *PSMC4* and mitochondrial ribosomal protein L19, *MRPL19*), positive and negative controls. All processes of hybridization, capture, cleanup, and digital data acquisition were performed with nCounter Prep Station^®^ and Digital Analyzer^®^ (NanoString Technologies, Seatle, WA, USA) according to the manufacturer’s instructions. Reporter counts were collected with the nsolver analysis software version 2.6. Samples were considered not evaluable if the geometrical mean (geomean) of counts corresponding to the housekeeping genes was lower than 100. Counts from *MET* probes were normalized in two steps, as described [31], and subjected to a logarithmic transformation to obtain the so‐called log‐*MET* expression values. Two cutoff values were used for log‐*MET* results; (a) the mean plus standard deviation (SD) was used to define cases with moderately (mod.)‐elevated *MET* mRNA, (b) the mean plus two SD for very‐high *MET* mRNA levels. Regarding *MET*Δex14 testing, log‐ratios were obtained dividing the normalized counts of the *MET*Δex14 probe by the normalized counts for the *MET*‐wt probe. The cutoff for *MET*Δex14 positivity was established as the average log‐ratio of the sample cohort plus 2 SD. Samples with no counts for the *MET*Δex14 probe were directly considered negative.

### 2.6 Validation of nCounter for detection of *MET* alterations

Using frozen pellets from Hs746T or E98 cells, we found that 5000 cells and 25 ng of RNA were sufficient for successful detection of spliced transcripts or very‐high MET mRNA levels, respectively. In contrast, pellets containing 500 000 of PC9 cells (*MET*‐wt) tested negative. Next, using FFPE blocks prepared by spiking different numbers of *MET*‐dependent cells (Hs746T and E98) in a suspension of PC9 cells, we established that 1 mm^2^ of a 4‐µm section with a minimum of 10% of Hs746T cells was required for *METΔex14* detection, whereas 30% of E98 cells were needed to detect high levels of *MET* mRNA expression by nCounter. In similar experiments, RT–PCR detected *METΔex14* mRNA in mixtures with 0.1% mutant tumor cells. Finally, we performed repeatability studies using FFPE tumors from advanced NSCLC patients. In the case of *METΔex14* detection by nCounter, four positive and 20 negative samples were analyzed in two independent experiments showing a 100% concordance. Regarding *MET* mRNA expression, 29 samples run in two independent experiments revealed concordant results in the classification for 28 of them (96.6%, CI = 82.8–99.4).

## 3 Results

### 3.1 Clinical samples

**Fig. 1:**
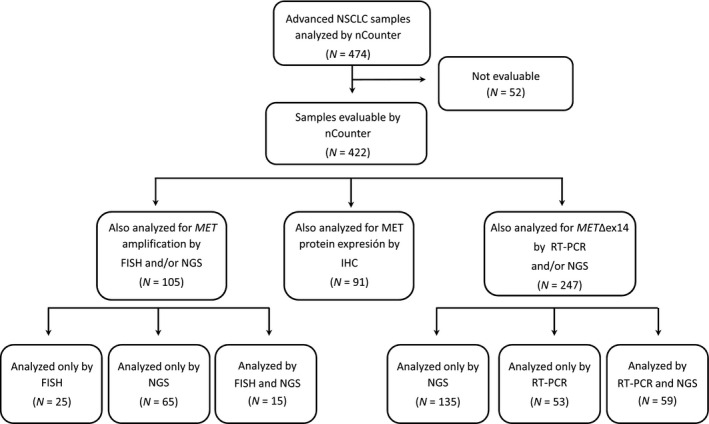
Flowchart of the study. FISH, fluorescence in situ hybridization; IHC, immunohistochemistry; NGS, next‐generation sequencing; NSCLC, non‐small‐cell lung cancer; RT–PCR, reverse transcription polymerase chain reaction.

A total of 474 FFPE NSCLC tumor samples were profiled using nCounter. Among them, 52 (11%) had geomean of housekeeping gene counts below 100 and were excluded from the study. The remaining 422 evaluable samples, corresponding to 405 patients, were mostly stage IIIB/IV adenocarcinomas (Table S4, Table 1). Four additional techniques were used in different subsets of samples to validate the MET status determined by nCounter. Copy number alterations were analyzed by FISH and/or NGS, *MET*Δex14 transcripts by RT–PCR or DNA‐based NGS, and protein expression by IHC (Fig. 1).

**Table 1:** Characteristics of all patients with valid results, patients positive for *MET*Δex14 by nCounter, and patients with very‐high and moderately elevated *MET* mRNA levels, also by nCounter, *N* (%).

Characteristics	All patients *N* = 405	*MET*Δex14 *N* = 13	*MET* mRNA very‐high *N* = 15	MET mRNA mod‐elevated *N* = 36
Gender				
Male	248 (61.2)	4 (30.8)	10 (66.7)	19 (52.8)
Female	146 (36.1)	9 (69.2)	5 (33.3)	16 (44.4)
Unknown	11 (2.7)	0 (0)	0 (0)	1 (2.8)
Age at diagnosis				
Median	63	70	58	64
Range	31–89	57–84	55–61	31–84
Smoking status				
Never	77 (19.0)	5 (38.5)	1 (6.7)	15 (41.7)
Former	134 (33.1)	2 (15.4)	6 (40.0)	7 (19.4)
Current	115 (28.4)	0 (0)	6 (40.0)	9 (25.0)
Unknown	79 (19.5)	6 (46.1)	2 (13.3)	5 (13.9)
Sample collection time				
No data	62 (15.3)	3 (23.1)	2 (13.3)	4 (11.1)
Baseline	294 (72.6)	10 (76.9)	9 (60.0)	27 (75.0)
Baseline and progression	7 (1.7)	0	0	0
Progression only	42 (10.4)	0	4 (27.8)	5 (13.9)

### 3.2 Detection of *MET*∆ex14 mRNA by nCounter in clinical samples

**Fig. 2:**
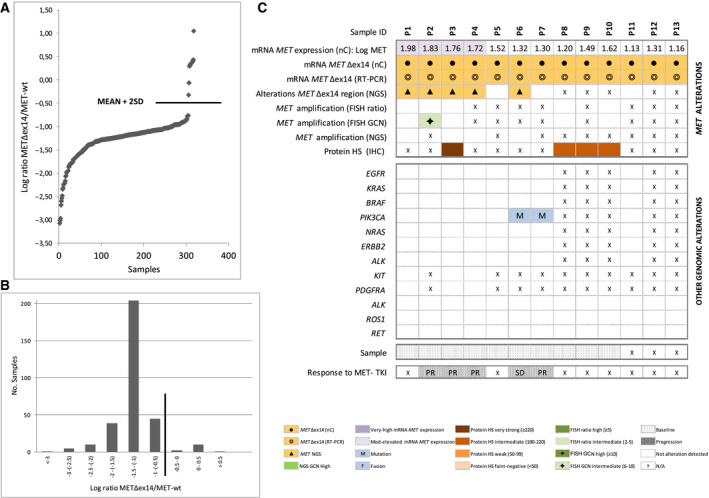
Detection of *MET*Δex14 by nCounter. (A) *MET*Δex14*/MET*‐wt normalized counts obtained by nCounter, expressed as log‐ratios. Only samples with detectable counts for *MET*Δex14 are plotted. The line indicates the cutoff for positivity (mean + 2SD). (B) Plot showing the bimodal distribution of *MET*Δex14*/MET‐*wt nCounter log‐ratios in the cohort. The line indicates the cutoff for positivity (mean + 2SD). (C) Heatmap displaying *MET*Δex14‐positive samples by nCounter (*N* = 13) and correlative results for RT–PCR, mRNA expression (nC), *MET* mutations (NGS), copy number alterations (FISH or NGS), immunohistochemistry (IHC), and other co‐occurring driver alterations. Patient numbers are shown in the top row. FISH, fluorescence in situ hybridization; HS, histoscore; IHC, immunohistochemistry; Mod, moderately; N/A nonavailable data; nC, nCounter; NGS, next‐generation sequencing; PR, partial response; RT–PCR, reverse transcription polymerase chain reaction; SD, stable disease. [Correction added on 9 December 2020, after first online publication: Missing details in the figure were amended.]

For each of the 422 samples evaluable, we calculated the log‐ratio of the normalized nCounter counts corresponding to the *MET*Δex14 *vs*. the *MET‐*wt probes. A sample was considered positive if the log‐ratio was above a threshold value, established as the mean plus two times the SD of all samples analyzed (Fig. 2A). The *MET*Δex14*/ MET‐*wt log‐ratios in our cohort showed a bimodal distribution with the cutoff value separating the two populations (Fig. 2B). A total of 13 *MET*Δex14‐positive patients (3%) were identified, with a majority of nonsmoking females and a median age of 70 years (Table 1). Of them, five were treated with MET‐TKIs and showed partial responses (*N* = 4) or stabilization of the disease (*N* = 1) by RECIST criteria (Fig. 2C).

Eight of the 13 *MET*Δex14‐positive cases could be submitted to DNA‐based NGS. Mutations affecting exon 14 splicing sites were detected in five of them (62.5%), mainly deletions in the acceptor splice‐site region (Table S5). Regarding other alterations, two patients showed *PIK3CA* mutations while concomitant *MET* amplification by GCN (6–10) was identified in only one sample (20%). However, this case was negative (< 2) by *MET*/CEP7 ratio. Sufficient tissue to perform MET IHC was available for four of the 13 patients; MET staining was intermediate in three cases and strong in one (Fig. 2C).

### 3.3 Quantification of *MET* mRNA expression levels by nCounter in clinical samples

**Fig. 3:**
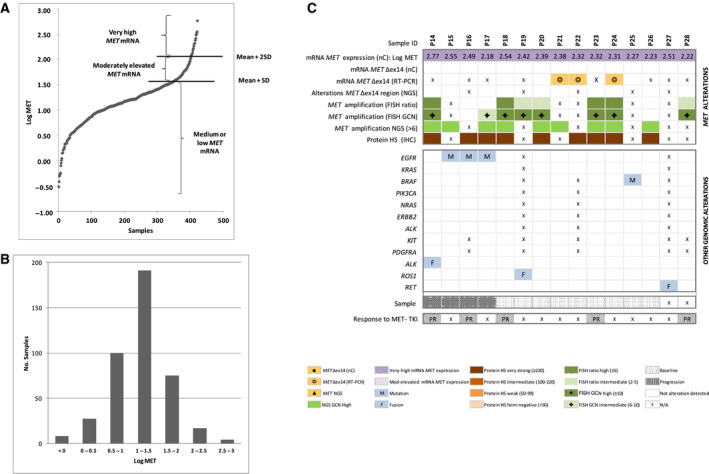
Quantification of *MET* mRNA expression levels by nCounter. (A) *MET* normalized counts obtained by nCounter, expressed as log‐*MET*. The cutoff values for moderately elevated *MET* mRNA expression (mean + SD) and very‐high *MET* mRNA expression (mean + 2SD) are indicated with lines. (B) Plot showing the unimodal distribution of log‐MET in the sample cohort. (C) Heatmap displaying samples with very‐high levels of *MET* mRNA expression by nCounter (*N* = 15) and corresponding results for *MET*Δex14 (nC), RT–PCR, *MET* mutations (NGS), copy number alterations (FISH or NGS), immunohistochemistry (IHC), and other co‐occurring driver alterations. Patient numbers are shown in the top row. FISH, fluorescence in situ hybridization; GCN, gene copy number; HS, histoscore; IHC, immunohistochemistry; Mod, moderately; N/A nonavailable data; nC, nCounter; NGS, next‐generation sequencing; PR, partial response; RT–PCR, reverse transcription polymerase chain reaction; SD, stable disease

**Fig. 4:**
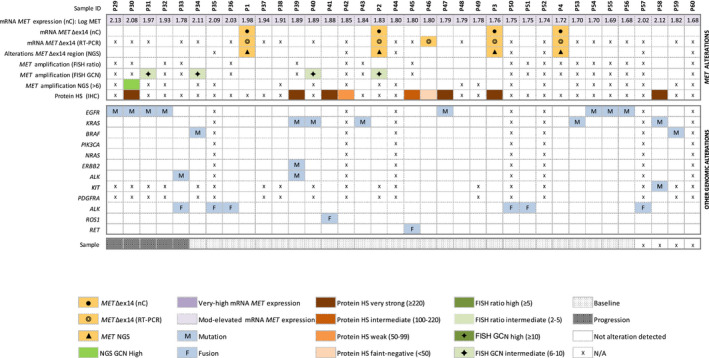
Heatmap displaying samples with mod‐elevated levels of *MET* mRNA expression by nCounter (*n* = 36). Corresponding results for *MET*Δex14 (nC) RT–PCR, *MET* mutations (NGS), copy number alterations (FISH or NGS), immunohistochemistry (IHC), and other co‐occurring driver alterations are also shown. Patient numbers are shown in the top row. FISH, fluorescence in situ hybridization; GCN, gene copy number; HS, histoscore; IHC, immunohistochemistry; Mod, moderate; N/A nonavailable data; nC, nCounter; NGS, next‐generation sequencing; RT–PCR, reverse transcription polymerase chain reaction.

The raw counts of the *MET* probes for each sample were transformed into logarithmic normalized data (log‐MET). According to our classification algorithm (see Section 2), 15/422 samples (3.5%) presented very‐high *MET* mRNA levels (Fig. 3) and 36/422 samples (8.5%) mod‐elevated *MET* mRNA levels by nCounter (Fig. 4). In contrast to the log‐ratio, the distribution of the log‐MET values was unimodal, although a Kolmogorov–Smirnov test revealed a significant deviation from normality (*P = *0.001; Fig. 3A,B). Most of the 15 patients with very‐high *MET* mRNA levels were males, former or current smokers with a median age of 58 years (Table 1). They invariably showed very strong IHC staining (≥ 220), tested negative for *MET*Δex14 by nCounter and 92% (11/12) had *MET* amplification by FISH or NGS (Fig. 3C). Finally, among the 9 baseline patients with very‐high *MET*, no other driver was detected in seven (77.8%). Five patients with very‐high *MET* mRNA were treated with MET‐TKIs, all of them showed partial responses by RECIST criteria. Interestingly, one was negative by FISH (Fig. 3C).

Regarding the 36 cases with mod‐elevated *MET* mRNA levels, we found comparable numbers of males and females, smokers, and never smokers with median age of 64 years (Table 1). The majority (19/27) of baseline samples with mod‐elevated MET and genotyping data available harbored concurrent genetic alterations, being mutations in KRAS proto‐oncogene GTPase (*KRAS*) and epidermal growth factor receptor (*EGFR*), fusions in ALK receptor tyrosine kinase (*ALK*), and *MET*Δex14 the most prevalent (*N* = 4 each; Fig. 4).

### 3.4 **Comparison of *METΔex14* nCounter results with RT**–**PCR and DNA‐based NGS in clinical samples**

The concordance of nCounter with RT–PCR and DNA‐based NGS for the detection of *MET*Δex14 is shown in Table S6, together with the corresponding values of specificity, sensitivity, and Cohen’s kappa. We observed a substantial agreement when comparing nCounter *vs*. DNA‐based NGS, with a 98.5% concordance rate (CI = 95.6–99.5, Cohen’s kappa 0.76) and only three discordant samples, all of them positive by nCounter and negative by DNA‐based NGS. Regarding nCounter and RT–PCR, there was a fair agreement (90.2%, CI = 83.3–94.4, Cohen’s kappa 0.65) and all the discordant cases (*N* = 11) were negative by nCounter and positive by RT–PCR. Six of those discordant samples had been analyzed by DNA‐based NGS; mutations associated with *MET*Δex14 were not detected in any case (Fig. S1). Five of them with remaining material were further investigated by an orthogonal RNA‐based NGS and all tested negative for *MET*Δex14 skipping transcripts. Finally, we systematically sequenced the 106 bp cDNA band, corresponding to the *MET*Δex14 mRNA, obtained in RT–PCR‐positive samples. No differences were observed between concordant and discordant samples, being the base sequence of the exon 13–exon 15 junction identical in all cases to the sequence described in the literature [9].

### 3.5 Comparison of *MET* expression levels by nCounter with *MET* amplification by FISH and NGS in clinical samples

A total of 40 samples had evaluable data by FISH and nCounter. Three different criteria for *MET* positivity were used for FISH evaluation (see Section 2). A moderate to substantial agreement was observed if the nCounter very‐high cutoff was employed, with the highest agreement for the FISH ratio *MET*/CEP7 ≥ 2 (concordance rate 92.5%, Cohen’s kappa 0.778; Table S7). The only three discordant samples were positive by nCounter and negative by FISH. Two of them had remaining material available and were submitted to RNA‐based NGS, testing negative for known *MET* gene fusions. If the nCounter mod‐elevated cutoff was selected, the agreement was only fair with any of the three FISH amplification criteria, with concordance rates of 50%‐70% and Cohen’s kappa 0.185–0.410 (Table S8).

DNA‐based NGS with the GeneRead platform, which can detect amplifications in several genes, was performed in 80 samples with nCounter data. *MET* amplification by DNA‐based NGS showed an almost perfect agreement with very‐high *MET* mRNA levels, with a Cohen’s kappa of 0.886 and a 97.5% concordance rate (Table S7). The only two discordant samples were *MET* amplified by NGS but did not show very‐high *MET* mRNA expression levels by nCounter. In contrast, if mod‐elevated *MET* mRNA levels were employed, the agreement with *MET* amplification by NGS was significantly worse (Cohen’s kappa 0.494) and the 14 discordant cases had moderately elevated *MET* mRNA but did not show copy number gains (Table S8).

### 3.6 Comparison of MET expression levels by nCounter and IHC in clinical samples

Ninety‐one samples were used to perform a comparative study of IHC *vs*. nCounter for the quantification of *MET* expression levels. Among these 91 samples, 34 showed very strong MET by IHC (HS ≥ 220), 19 intermediate (HS 100–220), 21 weak (HS 50–99), and 17 were very weak‐negative (HS < 50). In general, a fair agreement was observed between IHC and nCounter expression levels, with IHC positivity observed among cases with both mod‐elevated and very‐high mRNA levels (Tables S7 and S8).

### 3.7 Expression levels of *MET* mRNA in clinical samples with driver alterations

Finally, we compared *MET* mRNA levels by nCounter in patients harboring different drivers (Fig. S2). Baseline *MET* expression by nCounter was found to be significantly increased in samples with *MET* amplification, *MET*Δex14, and *BRAF* mutations (*P* < 0.05 in a Mann–Whitney U test), but not in cases with *EGFR* and *KRAS* mutations or *ALK* rearrangements. Next, we compared *MET* mRNA expression in samples at baseline *vs*. progression. Overall, there were no significant differences in log‐*MET* values between the 306 basal and the 52 progression samples included in the study. When classified by drivers, we did observe a significant increase in *MET* mRNA levels in tumors at progression *vs*. baseline only in *EGFR*‐mutant samples. However, a subpopulation of samples at progression with high *MET* mRNA expression was apparent not only in *EGFR* mutant, but also in *ALK*‐positive rebiopsies.

## 4 Discussion

**Fig. 5:**
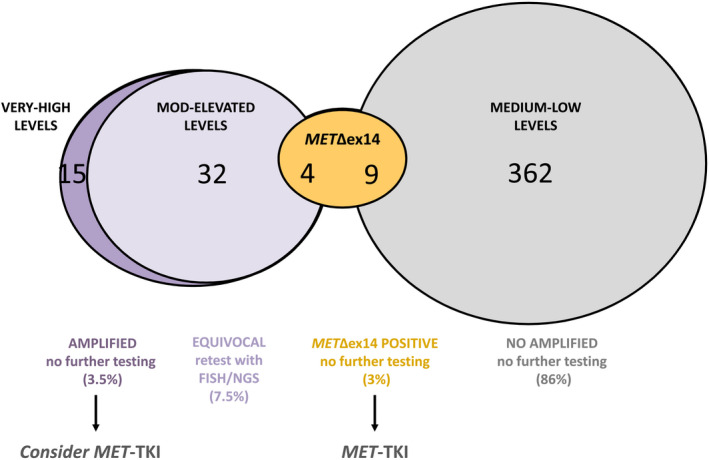
Graphical summary of the results obtained with nCounter. The numbers and percentages of evaluable samples with different *MET* mRNA expression are represented (*N* = 422). FISH, fluorescence in situ hybridization; *MET*∆ex14, *MET* exon 14 skipping mutation; Mod, moderately; TKI, tyrosine kinase inhibitor.

In this study, we comprehensively characterized the biologically relevant *MET* alterations—amplification and exon 14 skipping—in a large cohort of 474 NSCLC samples using of a RNA‐based technology (nCounter; Fig. 5) and we systematically compared the results with other currently available methods for MET testing. We also propose an algorithm for the selection of patients to be considered for MET‐TKI treatment, based on the RNA‐based results.

Our prevalence of *MET*Δex14 by nCounter (3%) is in line with the literature [6, 7], particularly when other RNA‐based techniques such as RNA sequencing or quantitative RT–PCR were used [32, 33]. In our series, all cases positive for *MET*Δex14 transcripts by nCounter (*N* = 13) were also detected by RT–PCR; however, a large subset of samples positive by RT–PCR (*N* = 11) tested negative by nCounter. Five of these discordant samples could be submitted to an orthogonal RNA‐based NGS, which confirmed them as negative. In addition, most of them were found to harbor known drivers, particularly *KRAS*. Although RT–PCR is used in some laboratories to identify *MET*∆ex14 transcripts, these results suggest that it might not be the most adequate technique for this purpose. Low level of *MET*Δex14 transcripts can emerge by splicing ‘mistakes’ in the cell without translating in any genomic alteration with oncogenic relevance [34, 35, 36]. These splicing ‘mistakes’ would test positive by mRNA amplification (RT–PCR), but not by nCounter, DNA‐, or RNA‐based NGS.

DNA‐based NGS using commercially available panels is also employed to determine *MET*∆ex14. However, this technique has been reported to detect only 63% of literature‐described splicing mutations associated with *MET*Δex14 [37], and reanalysis of 232 pan‐negative samples by DNA‐based NGS using RNA sequencing revealed six (2.5%) *MET*Δex14‐positive cases previously missed [33]. In our cohort, among the eight tumors with *MET*Δex14 by nCounter that were submitted to DNA‐based NGS, three (37.5%) tested negative for mutations associated with *MET*Δex14 splicing sites. One of these discordant patients was treated with a MET‐TKI and derived clinical benefit (Patient 7, Fig. 2C). It is probable that at least some of these cases involved large genomic deletions that are difficult to detect on DNA‐based NGS assays [32]. Therefore, our results confirm that DNA‐based techniques might underestimate the detection of *MET*Δex14 alterations.

Consistent with other studies [6, 7, 13, 14, 15, 38], most of the *MET* ∆ex14 patients detected by nCounter were elderly females, with a considerable proportion of never smokers (38%; Table 1). In addition, as previously reported, *MET*∆ex14 rarely coexisted with other drivers and was associated with moderate but not high MET expression by IHC [6, 38]. Finally, we only detected MET amplification by FISH or NGS in one (14%) of the *MET*∆ex14‐positive cases, compared to the 8–30% reported in the literature using different detection techniques and thresholds [6, 7, 13, 15, 17]. The significance of *MET* amplification in the context of *MET*∆ex14 alterations is unclear, but recent data from a prospective trial [15, 16, 17] indicate that it does affect response to MET‐TKIs, endorsing *MET*∆ex14 as a truly separate driver in NSCLC. Indeed, all *MET*∆ex14 patients in our cohort treated with MET‐TKIs derived clinical benefit, regardless of *MET* amplification status (Patients 2, 3, 4, 6, 7; Fig. 2C).

In addition to the presence of *MET*∆ex14 transcripts, we analyzed wt *MET* mRNA and could identify different clusters of patients according to *MET* expression levels. The group with very‐high *MET* mRNA (3.5%) comprised a majority of males, former, or current smokers with a median age of 58 years. Interestingly, very‐high *MET* mRNA expression closely correlated with *MET* gene amplification by NGS or FISH and was mutually exclusive with *MET*Δex14. Therefore, it is not surprising that the phenotype of the patients with very‐high *MET* mRNA agrees with the characteristics previously reported in patients with high *MET* amplification (defined as FISH gene copy number, GCN ≥ 6 or ≥ 10) [16, 17, 39, 40]. Furthermore, all the five very‐high *MET* mRNA cases treated with MET inhibitors achieved partial responses; the majority of case baseline (7/9) did not harbor any detectable driver. In contrast, the cohort of patients with mod‐elevated *MET* mRNA expression did not correlate with any specific phenotype, being distributed independently of gender and smoking habits, and known drivers were detected in most cases. Although the biological oncogenic significance of *MET* amplification in NSCLC has been controversial [3, 41], our results endorse the concept of *MET* being a true driver in patients with very‐high expression mRNA levels. In contrast, we believe that *MET* is unlikely to play a clinically relevant role in tumors with mod‐elevated expression levels.

Several trials have recently evaluated the efficacy of MET‐TKIs in *MET*∆ex14‐positive NSCLC. Overall response rates of 32% have been reported for crizotinib, a nonselective inhibitor, compared to 46–68% and 40–55% in first or subsequent lines for the selective inhibitors capmatinib, tepotinib, and savolitinib [11, 13, 14]. In contrast, the results obtained so far in trials enrolling *MET*‐amplified patients are significantly worse, with overall response rate (ORR) 20–47% [16, 17, 20, 39, 40, 42]. It is unclear whether the variety of responses observed in trials of MET‐TKIs may partly underlie a heterogeneous disease population with distinct sensitivities to MET‐TKIs and/or be a consequence of the different thresholds, methodologies, and scoring systems (*MET*/CEP7 ratios or GCN) that have been used as eligibility criteria.

Our study can shed some light on this relevant issue, and the characterization of *MET* expression levels in NSCLC here presented provides a unique opportunity to advance in the understanding of the processes underlying *MET* biology. For instance, our RNA‐based assessment allowed the identification of amplification‐negative tumors with very‐high *MET* expression levels and also the relatively infrequent cases with gene amplification that did not express *MET* mRNA. Remarkably, one of the patients with very‐high *MET* mRNA levels but negative by FISH (using the two usual cutoff values) was treated with a *MET* inhibitor achieving a partial response. These observations suggest that FISH positivity might be capturing a heterogeneous group regarding dependence on aberrant *MET* signaling and that RNA‐based techniques could improve the performance of FISH for patient selection. This, in turn, may help to explain the inferior response rates observed with MET‐TKIs in FISH‐amplified tumors. In order to improve outcomes to MET inhibition in this setting, it would be important to further investigate the efficacy of *MET*‐TKI in those tumors with gene amplification that do not express *MET* mRNA, and the real clinical value of high expression levels in amplification‐negative tumors where MET inhibition therapy could be considered.

Regarding *MET*Δex14, recent studies have suggested that MET protein expression is required for clinical benefit from MET‐TKIs [43]. Interestingly, the only *MET*Δex14 patient in our cohort who exhibited stable disease to MET‐targeted therapy had low levels of mRNA by nCounter, whereas all treated patients with mod‐elevated levels had partial responses. Whether mRNA expression levels can assist to predict outcome in patients with *MET*Δex14 is currently unknown but also merits further investigation. One of the limitations of our study derives from the fact that MET is known to be expressed not only in tumor cells but also in normal epithelial [44], dendritic, and other immune cells [45]. In consequence, particularly in cases with important stroma and/or inflammatory component, the mRNA expression levels obtained by nCounter might reflect the level of MET expression in the whole tumor rather than only in cancer cells. However, the good agreement observed between *MET* mRNA levels by nCounter and MET IHC staining in tumor cells indicated that, in most samples, the contribution of noncancer components to the nCounter results was not significant.

## 5 Conclusions

We have comprehensively characterized *MET* in a large cohort of advanced NSCLC and have validated the use of nCounter to identify *MET*Δex14 in this malignancy. Our work also provides useful insights into the biology of MET as a driver in NSCLC, supporting MET very‐high mRNA expression as a surrogate of amplification, and suggesting the relevance of MET mRNA levels in patients responding to MET‐TKIs. Our results support the use of mRNA‐based techniques for multiplex, accurate, and reliable assessment of *MET* alterations in order to select patients for MET‐targeted therapies.

## Conflict of interest

CT has received fees for consultancy/advisory roles from Pfizer, Novartis, MSD, Roche, AstraZeneca, and Takeda, and research funding from Pfizer and Novartis. CC has received travel grants from MSD, Pierre‐Fabre Oncology, fees for consultancy/advisory from Boehringer Ingelheim, Roche, Pfizer, and Boehringer Ingelheim. LLV has received fees for consultancy/advisory roles from AstraZeneca and Roche. SV has received fees for consultancy/advisory roles from Bristol‐Myers Squibb, Roche, MSD, Abbvie, Ose Pharma, and Merck. AAH has received fees for consultancy/advisory roles from Bristol‐Myers Squibb, Roche, MSD, and Lilly. MGC has received fees for consultancy/advisory roles from Bristol‐Myers Squibb, Roche, AstraZeneca, and Pierre‐Fabre Oncology, and research funding from AstraZeneca. AP has received fees for consultancy/advisory roles from Pfizer, Lully, Nanostring Technologies, Amgen, Oncolytics Biotech, Daiichi Sankyo, PUMA, Bristol‐Myers Squibb, Novartis, and Daiichi Sankyo, and research funding from Pfizer, Amgen, Roche, Novartis, and Daiichi Sankyo. NR has received fees for consultancy/advisory roles from MSD, BMS and Pfizer, and research funding from Pfizer, Novartis, and Ministry of Health, Instituto de Salud Carlos III, Spain. CA, RR, RRe, AGC, EM, NV, SC, SM, AA, IS, EA, SR, APR, IM, AFC, RP, CS, MMG, MDL, AMB, EG, WPJL, RR, LMM, and MAMV have declared no conflicts of interest.

## Author contributions

CA, CT, RR, RRe, AGC, EM, MAMV, and NR conceived and designed the study. CA, CT, RR, RRe, AGC, EM, SM, AA, EA, SR, MAMV, and NR conducted the experiments, acquired, and analyzed data. CC, NV, SC, SM, LLV, IS, IM, SV, AFC, RP, CS, MMG, MDL, AAH, AMB, MGC, EG, WPJL, RRo, LMM, and AP provided clinical samples and collected clinical information. NR, CT, CA, and MAMV wrote the manuscript. All authors reviewed and/or revised the manuscript.

### Peer Review

The peer review history for this article is available at https://publons.com/publon/10.1002/1878‐0261.12861.

## Supporting information


**Fig. S1.** Heatmap displaying the molecular and clinical characteristics samples with *MET*Δex14 discordant results between RT‐PCR and nCounter.
**Fig. S2.** *MET* mRNA expression levels by nCounter in samples harboring different driver alterations.
**Table S1.** MET amplification criteria used in clinical trials of MET inhibitors.
**Table S2.** Description of the NGS panels used in the study.
**Table S3.** Description of the nCounter codeset used in the study and probes design for *MET* wild type and *MET*Δex14.
**Table S4.** Characteristics of the samples with valid results, *N* (%). Fusions were determined by nCounter, mutations by NGS.
**Table S5.** Results for *MET* exon 14 alteration splice‐site regions and mutation types.
**Table S6.** Concordance of nCounter with RT‐PCR and NGS for *METex*Δ14 status.
**Table S7.** Concordance of the nCounter categorization using the cut‐off for very high *MET* levels with IHC, FISH and NGS.
**Table S8.** Concordance of the nCounter categorization using the cut‐off for moderately elevated *MET* levels with IHC, FISH and NGS.

## Data Availability

All data generated or analyzed during this study are included in this manuscript and its supplementary information files or are available from the authors upon reasonable request.
