# Supplementary material for: Multiplex RNA‐based detection of clinically relevant MET alterations in advanced non‐small cell lung cancer
Source: Mol Oncol. 2020 Dec 7;15(2):350–63. doi: 10.1002/1878-0261.12861 (PMC7858100; doi:10.1002/1878-0261.12861)
Supplement: Supplementary file 1 — Fig. S1. Heatmap displaying the molecular and clinical characteristics samples with METΔex14 discordant results between RT‐PCR and nCounter. Fig. S2. MET mRNA expression levels by nCounter in samples harboring different driver alterations. Table S1. MET amplification criteria used in clinical trials of MET inhibitors. Table S2. Description of the NGS panels used in the study. Table S3. Description of the nCounter codeset used in the study and probes design for MET wild type and METΔex14. Table S4. Characteristics of the samples with valid results, N (%). Fusions were determined by nCounter, mutations by NGS. Table S5. Results for MET exon 14 alteration splice‐site regions and mutation types. Table S6. Concordance of nCounter with RT‐PCR and NGS for METexΔ14 status. Table S7. Concordance of the nCounter categorization using the cut‐off for very high MET levels with IHC, FISH and NGS. Table S8. Concordance of the nCounter categorization using the cut‐off for moderately elevated MET levels with IHC, FISH and NGS. [file MOL2-15-350-s001.docx]

**Supplementary Information**

**RNA-based testing for multiplex detection of clinically relevant *MET* alterations in advanced non-small cell lung cancer. Cristina Aguado *et al*.**

**INDEX**

**CENTERS PARTICIPATING IN THE STUDY…………………………………....…..3**

**SUPPLEMENTARY TABLES……………………...…………………………………….4**

**SUPPLEMENTARY FIGURES…………………………………………………………11**

**CENTERS PARTICIPATING IN THE STUDY**

| **Hospital** | **City** |
| --- | --- |
| Hospital Clínic | Barcelona |
| Hospital Quirón Dexeus | Barcelona |
| Hospital General de Granollers | Granollers |
| Hospital de la Santa Creu i Sant Pau | Barcelona |
| Institut Català d’Oncologia | Hospitalet de Llobregat |
| Hospital General de Catalunya | Sant Cugat del Vallès |
| Hospital Sagrat Cor | Barcelona |
| Centro Médico Teknon | Barcelona |
| Clinica Universidad de Navarra /Center for Applied Medical Research (CIMA) | Pamplona |
| Clínica del Country | Bogotá |

**SUPPLEMENTARY TABLES**

**Table S1: MET amplification criteria used in clinical trials of MET inhibitors**

| **Clinical trial** | **MET inhibitor** | **CRITERIA for MET amplification** | **References** |
| --- | --- | --- | --- |
| NCT03911193  (CABinMET) | Cabozantinib | MET/CEP7 ≥2.2 | [1] |
| NCT02435121 | SAR125844 | GCN >4 in ≥10% cells and MET/CEP7 ≥2 | [2] |
| NCT00585195 | Crizotinib | MET/CEP7 ≥1.8 | [3] |
| NCT02648724 | Sym015 humanized antibodies | MET/CEP7 >2.2 | [4] |
| NCT02499614  (METROS) | Crizotinib | MET/CEP7 >2.2 | [5] |
| NCT01324479 | Capmatinib | MET/CEP7 >2 and GCN ≥5 (initial criteria)  CGN ≥ 6 (final, published) | [6, 7] |
| NCT02414139  (GEOMETRY mono-1) | Capmatinib | GCN < 4, GCN ≥ 4 and < 6 and CGN ≥ 6 (initial criteria)  GCN>10 (final, published) | [8] |
| CL1-49076-003 | S49076 | MET/CEP7 ≥ 2.0  GCN ≥ 6  ≥ 10% cells with ≥ 15 copies  ≥ 50% cells with ≥ 5 copies | [9] |

**Table S2: Description of the NGS panels used in the study**

| **NGS panel** | **Genes included** |
| --- | --- |
| Oncomine^TM^ Solid Tumour | Mutations: *AKT1, ALK, BRAF, CTNNB1, DDR2, EGFR, ERBB2/HER2, ERBB4, FBXW7, FGFR1, FGFR2, FGFR3, MET, KRAS, PIK3CA, PTEN, NRAS, MAP2K1, NOTCH1, SMAD4, STK11, TP53* |
| GeneRead^TM^ QIAact Lung DNA UMI | Mutations: *AKT1, ALK, BRAF, DDR2, EGFR, ERBB2/HER2, ESR1, FGFR1, KIT, KRAS, MAP2K1, MET, NRAS, NTRK1, PDGFRA, PIK3CA, PTEN, ROS1, RICTOR*  Amplifications: *EGFR, FGFR1, ERBB2/HER2, MET, RICTOR* |
| GeneRead^TM^ QIAact RNA Fusion UMI | Fusions: *BRAF, ALK, ROS1, RET, MET, NRG1, RAF, FGFR1, FGFR3, NTRK1, KRAS*  Splicing variant: *MET*Δex14 |

**Table S3:**  **Description of the nCounter codeset used in the study and probes design for *MET* wild type and *MET*Δex14**

| **Probes** | **Number** |
| --- | --- |
| Gene Fusions Probes (*ALK, ROS1, RET, NTRK1*) | 29 pairs |
| Imbalance Probes (*ALK, ROS1, RET, NTRK1*) | 32 pairs |
| Housekeeping Genes Probes (*ACTB, PSMC4, MRPL19*) | 3 pairs |
| MET Gene Probes (*MET*Δex14*, MET wild type**) | 2 pairs |

**MET*Δex14, accession number RCC_AS01_065.1, target sequence TCCTGTGGCTGAAAAAGAGAAAGCAAATTAAAGATCAGTTTCCTAATTCATCTCAGAACGGTTCATGCCGACAAGTGCAGTATCCTCTGACAG

***MET* wild type;, accession number RCC_AS01_066.2, target sequence CCTGTGGCTGAAAAAGAGAAAGCAAATTAAAGATCTGGGCAGTGAATTAGTTCGCTACGATGCAAGAGTACACACTCCTCATTTGGATAGG

**Table S4:** **Characteristics of the samples with valid results, *N* (%). Fusions were determined by nCounter, mutations by NGS**

| **Characteristics** | **All samples (*N*=422)** |
| --- | --- |
| **Histological type** |  |
| Adenocarcinoma | 299 (70.9) |
| Squamous cell carcinoma | 45 (10.7) |
| Pleomorphic | 6 (1.4) |
| Other | 30 (7.1) |
| Unknown | 42 (9.9) |
| **UICC stage** |  |
| I-IIIA | 59 (13.9) |
| IIIB-IV | 286 (67.8) |
| Unknown | 77 (18.3) |
| **Collection time** |  |
| Basal | 306 (72.5) |
| Progression | 52 (12.3) |
| Unknown | 64 (15.2) |
| **Genetic alterations detected** |  |
| *ALK* fusion | 58 (13.8) |
| *ROS1* fusion | 5 (1.2) |
| *RET* fusion | 4 (0.9) |
| *MET*Δex14 | 13 (3.1) |
| *EGFR* mutations | 32 (7.6) |
| *KRAS* mutations | 65 (15.4) |
| *BRAF* mutations | 12 (2.9) |
| Other alterations | 21 (4.9) |
| None | 212 (50.2) |

**Table S5:** **Results for *MET* exon 14 alteration splice-site regions and mutation types**

| Patient ID | *MET*Δex14 Intron | *MET*Δex14  Splice-site Region | *MET*Δex14 Mutation Type | *MET*Δex14 alteration |
| --- | --- | --- | --- | --- |
| P1 | 13 | Acceptor | Deletion | c.2942-6_2963delTTTAAGATCTG  GGCAGTGAATTAGTTCG |
| P2 | 13 | Acceptor | Deletion | c.2942-20_2942-9del |
| P3 | 13 | Acceptor | Deletion | c.2942-52_2988delGGGGCCCATGAT  AGCCGTCTTTAACAAGCTCTTTCTTTCTCTCTGTTTAAGATCTGGGCAGTGAATTAGTTCGCTACGATGCAAGAGTACACACTCCT |
| P4 | 14 | Donor | Deletion | c.3082_3082+1delGG |
| P6 | 13 | Acceptor | Deletion | c.2942-27_2942-16delAAGCTCTTTCTT |

**Table S6:** **Concordance of nCounter with RT-PCR and NGS for *METex*Δ14 status**

| **Variable** | ***MET*Δex14 status** | |
| --- | --- | --- |
| **Techniques**  **compared** | ***N* = 112** | ***N* = 194** |
|  | **nC *vs*. RT-PCR** | **nC *vs*. NGS** |
| Nº concordant samples | 101 | 191 |
| Nº discordant samples | 11 | 3 |
| Sensitivity | 54.2% (CI = 35.1-72.1) | 100% (CI = 56.6-100.0) |
| Specificity | 100% (CI = 94.7-100.0) | 98.4% (CI = 95.4-99.5) |
| Concordance | 90.2% (CI = 83.3-94.4) | 98.5% (CI = 95.6-99.5) |
| Cohen’s kappa | 0.650 (CI = 0.466-0.834) | 0.762 (CI = 0.502-1.000) |

Abbreviations: nC, nCounter; NGS, next generation sequencing; RT-PCR, reverse transcription polymerase chain reaction

**Table S7: Concordance of the nCounter categorization using the cut-off for very high *MET* levels with IHC, FISH and NGS.**

| **Variable** | **MET expression** | | ***MET* amplification** | | | |
| --- | --- | --- | --- | --- | --- | --- |
|  | ***N* = 91** | | ***N* = 40** | | | ***N = 80*** |
| **Techniques**  **compared** | **nC *vs*. IHC**  **(3+ in ≥ 50%)** | **nC *vs*. IHC**  **(HS ≥ 220)** | **nC *vs.* FISH**  **(r ≥ 2)** | **nC *vs.* FISH**  **(GCN≥ 6)** | **nC *vs.* FISH**  (**50%≥ 5 or 10%≥ 15)** | **nC *vs.* NGS** |
| Nº concordant samples | 78 | 83 | 37 | 34 | 31 | 78 |
| Nº discordant samples | 13 | 8 | 3 | 6 | 9 | 2 |
| Sensitivity | 41.0%  (CI = 23.3-61.3) | 52.9%  (CI = 31.0-73.8) | 100%  (CI = 64.6-100.0) | 66.7%  (CI = 88.3-100.0) | 52.6%  (CI = 31.7-72.7) | 81.8%  (CI = 52.3-94.9) |
| Specificity | 100%  (CI = 94.7-100.0) | 100%  (CI = 95.1-100.0) | 90.9%  (CI = 76.4-96.9) | 92.9%  (CI = 77.4-98.2) | 100%  (CI = 84.5-100.0) | 100%  (CI = 94.7-100.0) |
| Concordance | 85.7%  (CI = 77.1-91.5) | 91.2%  (CI = 83.6-95.5) | 92.5%  (CI = 80.1-97.4) | 92.9%  (CI = 77.4-98.0) | 77.5%  (CI = 62.5-87.7) | 97.5%  (CI = 91.3-99.3) |
| Cohen’s kappa | 0.513  (CI = 0.299-0.727) | 0.647  (CI = 0.428-0.855) | 0.778  (CI = 0.542-1.000) | 0.625  (CI = 0.355-0.895) | 0.538  (CI = 0.303-0.774) | 0.886  (CI = 0.731-1.000) |

Abbreviations: FISH, fluorescence in situ hybridization; HS, histoscore; IHC, immunohistochemistry; nC, nCounter; NGS, next generation sequencing

**Table S8:** **Concordance of the nCounter categorization using the cut-off for moderately elevated *MET* levels with IHC, FISH and NGS.**

| **Variable** | **MET expression** | | ***MET* amplification** | | | | |
| --- | --- | --- | --- | --- | --- | --- | --- |
|  | ***N* = 91** | | ***N* = 40** | | |  | ***N* = 80** |
| **Techniques**  **compared** | **nC *vs*. IHC**  **(3+ in ≥ 50%)** | **nC *vs*. IHC**  **(HS ≥ 220)** | **nC *vs.* FISH**  **(r ≥ 2)** | **nC *vs.* FISH**  **(GCN≥ 6)** | **nC *vs.* FISH**  (**50%≥ 5 or 10%≥ 15)** |  | **nC *vs.* NGS** |
| Nº concordant samples | 79 | 86 | 20 | 25 | 28 | 66 | |
| Nº discordant samples | 12 | 5 | 20 | 15 | 12 | 14 | |
| Sensitivity | 63.6%  (CI = 43.0-80.3) | 88.2%  (CI = 65.7-96.7) | 100%  (CI = 64.6-100) | 100%  (CI = 75.8-100) | 89.5%  (CI = 68.6-97.1) | 90.9%  (CI = 62.3-98.4) | |
| Specificity | 94.2%  (CI = 86.0-97.7) | 95,9%  (CI = 88.7-98.6) | 39.4%  (CI = 24.7-56.3) | 46,4%  (CI = 29.5-64.2) | 52.4%  (CI = 32.4-71.7) | 81.2%  (CI = 70.4-88.7) | |
| Concordance | 86.8%  (CI = 78.3-92.3) | 94.5%  (CI = 87.8-97.6) | 50.0%  (CI = 35.2-64.8) | 62.5%  (CI = 47.0-75.8) | 70.0%  (CI = 54.6-81.9) | 82.5%  (CI = 72.7-89.3) | |
| Cohen’s kappa | 0.617  (CI = 0.421-0.812) | 0.823  (CI = 0.673-0.973) | 0.185  (CI = 0.04-0.331) | 0.342  (CI = 0.144-0.540) | 0.410  (CI = 0.153-0.668) | 0.494  (CI = 0.281-0.708) | |

Abbreviations: FISH, fluorescence in situ hybridization; GCN, gene copy number**;** HS, histoscore; IHC, immunohistochemistry;; nC, nCounter; NGS, next generation sequencing; r, ratio *ME*

**SUPPLEMENTARY FIGURES**

**
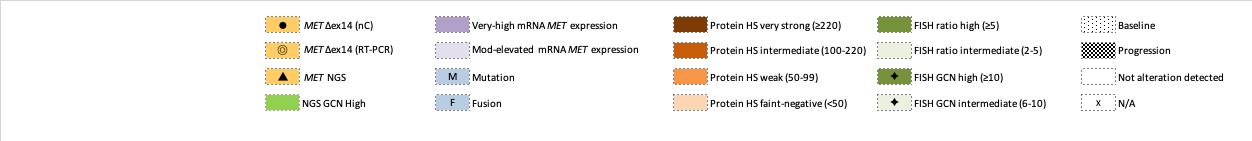
**

**Figure S1:** **Heatmap displaying the molecular and clinical characteristics samples with *MET*Δex14 discordant results between RT-PCR and nCounter.** Patient numbers are shown in the top row. Abbreviations: Mod, moderate; FISH, fluorescence in situ hybridization; NGS, next-generation sequencing; IHC, immunohistochemistry; nC, nCounter; HS, histoscore; RT-PCR, reverse transcription polymerase chain reaction; GCN, gene copy number; N/A non-available data.

**Figure S2: *MET* mRNA expression levels by nCounter in samples harboring different driver alterations.** **(A)** *MET* mRNA expression levels at presentation. **(B)** Comparison of *MET* mRNA expression levels of basal samples (b) *vs.* samples at progression (p).

**SUPPLEMENTARY REFERENCES**

[1] M. D'Arcangelo, D. Tassinari, F.D. Marinis, A. Delmonte, D. Galetta, F. Cecere, S. Pilotto, F. Zanelli, L. Bonanno, L. Landi, F. D'Incà, F. Cappuzzo, P2.01-15 Phase II Single Arm Study of CABozantinib in Non-Small Cell Lung Cancer Patients with MET Deregulation (CABinMET), Journal of Thoracic Oncology, 14 (2019) S644.

[2] E. Angevin, G. Spitaleri, J. Rodon, K. Dotti, N. Isambert, S. Salvagni, V. Moreno, S. Assadourian, C. Gomez, M. Harnois, A. Hollebecque, A. Azaro, A. Hervieu, K. Rihawi, F. De Marinis, A first-in-human phase I study of SAR125844, a selective MET tyrosine kinase inhibitor, in patients with advanced solid tumours with MET amplification, Eur J Cancer, 87 (2017) 131-139.

[3] Camidge DR, Otterson GA, Clark JW, Ou I, Weiss J, Ades S, Conte U, Tang Y, Wang S, Murphy D, Wilner KD, V. LC, Crizotinib in patients (pts) with MET-amplified non-small cell lung cancer (NSCLC): Updated safety and efficacy findings from a phase 1 trial., Journal of Clinical Oncology, 36 (2018) S9062.

[4] Camidge DR, Janku F, Martinez-Bueno A, L.J. Catenacci D, L. S, Safety and preliminary clinical activity of the MET antibody mixture, Sym015 in advanced non-small cell lung cancer (NSCLC) patients with MET amplification/exon 14 deletion (METAmp/Ex14∆), Journal of Clinical Oncology, 38 (2020) S9510.

[5] L. Landi, R. Chiari, M. Tiseo, F. D'Inca, C. Dazzi, A. Chella, A. Delmonte, L. Bonanno, D. Giannarelli, D.L. Cortinovis, F. de Marinis, G. Borra, A. Morabito, C. Gridelli, D. Galetta, F. Barbieri, F. Grossi, E. Capelletto, G. Minuti, F. Mazzoni, C. Verusio, E. Bria, G. Ali, R. Bruno, A. Proietti, G. Fontanini, L. Crino, F. Cappuzzo, Crizotinib in MET-Deregulated or ROS1-Rearranged Pretreated Non-Small Cell Lung Cancer (METROS): A Phase II, Prospective, Multicenter, Two-Arms Trial, Clin Cancer Res, 25 (2019) 7312-7319.

[6] M. Schuler, R. Berardi, W.T. Lim, M. de Jonge, T.M. Bauer, A. Azaro, M. Gottfried, J.Y. Han, D.H. Lee, M. Wollner, D.S. Hong, A. Vogel, A. Delmonte, M. Akimov, S. Ghebremariam, X. Cui, N. Nwana, M. Giovannini, T.M. Kim, Molecular correlates of response to capmatinib in advanced non-small-cell lung cancer: clinical and biomarker results from a phase I trial, Ann Oncol, (2020).

[7] Y.J. Bang, W.C. Su, M. Schuler, D.H. Nam, W.T. Lim, T.M. Bauer, A. Azaro, R.T.P. Poon, D. Hong, C.C. Lin, M. Akimov, S. Ghebremariam, S. Zhao, M. Giovannini, B. Ma, Phase 1 study of capmatinib in MET-positive solid tumor patients: Dose escalation and expansion of selected cohorts, Cancer Sci, 111 (2020) 536-547.

[8] J. Wolf, T. Seto, J.Y. Han, N. Reguart, E.B. Garon, H.J.M. Groen, D.S.W. Tan, T. Hida, M. de Jonge, S.V. Orlov, E.F. Smit, P.J. Souquet, J. Vansteenkiste, M. Hochmair, E. Felip, M. Nishio, M. Thomas, K. Ohashi, R. Toyozawa, T.R. Overbeck, F. de Marinis, T.M. Kim, E. Laack, A. Robeva, S. Le Mouhaer, M. Waldron-Lynch, B. Sankaran, O.A. Balbin, X. Cui, M. Giovannini, M. Akimov, R.S. Heist, G.m.-. Investigators, Capmatinib in MET Exon 14-Mutated or MET-Amplified Non-Small-Cell Lung Cancer, N Engl J Med, 383 (2020) 944-957.

[9] Garzon -Ibanez M, Jordana-Ariza N, González-Cao M, Molina-Vila MA, Smutna V, Cattan V, Rosell R, S. Viteri-Ramirez, Abstract 2521: Molecular profiling of T790M-negative NSCLC patients progressing on EGFR-TKI enrolled in the CL1-49076-003 trial with a MET/AXL/FGFR inhibitor in combination with gefitinib, Cancer Research, 79 (2019) S2521.
